# Identification of Virulence Properties in *Salmonella* Typhimurium DT104 Using *Caenorhabditis elegans*

**DOI:** 10.1371/journal.pone.0076673

**Published:** 2013-10-04

**Authors:** Surasri N. Sahu, Yuda Anriany, Christopher J. Grim, Sungji Kim, Zenas Chang, Sam W. Joseph, Hediye N. Cinar

**Affiliations:** 1 Division of Virulence Assessment, Food and Drug Administration, Laurel, Maryland, United States of America; 2 Oak Ridge Institute for Science and Education, Oak Ridge, Tennessee, United States of America; 3 Department of Biological Sciences, Prince George’s Community College, Laurel, Maryland, United States of America; 4 Kyungpook National University (KNU), Daegu, South Korea; 5 Department of Cell Biology and Molecular Genetics, University of Maryland, College Park, Maryland, United States of America; Indian Institute of Science, India

## Abstract

*Salmonella enterica* serover Typhimurium definitive phage type DT104, resistant to multiple antibiotics, is one of the most widespread *Salmonella* species in human infection worldwide. Although several cohort studies indicate that DT104 carrying the multidrug resistance (MDR) locus on salmonella genomic island 1 is a possible hyper-virulent strain compared to DT104 strains without MDR, or other *Salmonella enterica* serotypes, existing experimental evidence regarding virulence properties associated with the MDR region is controversial. To address this question, we constructed an isogenic MDR deletion (∆MDR) mutant strain of DT104, SNS12, by allelic exchange and used *Caenorhabditis elegans* as a host model to assess differences in virulence between these two strains. SNS12 exhibited decreased virulence in *C. elegans*, and we observed increased colonization and proliferation of the intestine of *C. elegans* by DT104. The immune response against MDR-carrying DT104 appears to function through a non-canonical Unfolded Protein Response (UPR) pathway, namely prion-like-(QN-rich)-domain-bearing protein pathway (PQN), in a *ced-1* dependent manner in *C. elegans*. Further, we also demonstrate that genes of the PQN pathway and antimicrobial peptide gene *abf-2*, are expressed at higher transcriptional levels in worms immediately following exposure to DT104, in comparison with worms exposed to SNS12. Altogether, our results suggest that the MDR region of *Salmonella* Typhimurium DT104 has a direct role in virulence against *Caenorhabditis elegans.*

## Introduction

Non-typhoid *Salmonella enterica* is one of the primary causes of food-borne illness throughout the world [1]. Among more than 2,500 *Salmonella enterica* serovars, Typhimurium is the second most prevalent, behind Enteritidis, in human infection worldwide [2]. *Salmonella enterica* serovar Typhimurium definitive phage type DT104 (hereafter, DT104) [3], first isolated in the 1960s, emerged in the 1990s as many isolates of this strain were found to have acquired multidrug resistance, specifically to ampicillin, chloramphenicol, streptomycin, sulfonamides and tetracycline (ACSSuT) [4].

Salmonella genomic island 1 (SGI1) is a 43 kb genomic island containing 44 open reading frames (ORFs) [5]. The multidrug resistance (MDR) region of DT104 is localized to a 13 kb segment of SGI1 [3,5,6]. Several cohort studies have indicated that DT104 carrying the MDR region is a hyper-virulent strain, as compared to DT104 strains without MDR or other *Salmonella enterica* serotypes [7,8]. The enhanced virulence does not appear to be due to increased invasiveness, as no significant increase in the invasive properties of DT104 were observed when tested in tissue culture assays and a mouse model of systematic salmonellosis [9–11]. Conversely, insertional inactivation of the MDR locus in DT104 was reported to reduce virulence in chickens when compared with the isogenic parent strain [12].

The soil nematode, *C. elegans*, has been used as an invertebrate host model to identify and assess virulence factors of several human pathogens, including *Salmonella enterica* Typhimurium [13] [14] [15]. A short life cycle facilitates rapid genetic experiments and is one of the major advantages for researchers working with this organism. *C. elegans* eggs are fertilized within the adult hermaphrodite and laid a few hours afterward--at about the 40 cell stage. *C. elegans* embryos develop rapidly and hatch after 14 hours. The first larval stage is completed after another 12 hours and the animals proceed through four molt cycles (L1-L4) before becoming adults. When animals reach adulthood, each produce about 300 progeny over the course of 3-4 days. The life cycle is temperature-dependent; *C. elegans* goes through a reproductive life cycle, egg to egg-laying parent, in 5.5 days at 15°C, 3.5 days at 20°C, and 2.5 days at 25°C. At 22°C, *C. elegans* has an average life span of approximately 2–3 weeks and a generation time of approximately 4 days, under laboratory conditions.

The *C. elegans – Salmonella* Typhimurium host-pathogen interaction model was established more than a decade ago, and it provides a powerful system to understand the virulence mechanisms of the pathogen and the immune response of the host [16–18]. *Salmonella* Typhimurium has been shown to colonize and establish a persistent intestinal infection in *C. elegans*, even after limited exposure [16,18]. Several pathogen and host factors have been found to mimic higher-order organism animal models of infection. For example, a regulator of Salmonella virulence-related genes in vertebrates, the PhoP/PhoQ signal transduction system, has also been found to be involved in *Salmonella* Typhimurium pathogenesis in *C. elegans* [16]. Acid tolerance capacity of *Salmonella* contributes to the survival of the microorganism during its passage through the alimentary tract and subsequently protects against the acidic environment within lysosomes [19]. The *fur-1*, *ompR*, and *rpoS* genes of *Salmonella*, which are known to be involved in different aspects of acid tolerance in human infection, have also been found to be important in *C. elegans* infection [18]. Lipopolysaccharide (LPS) was also shown to be required for the induction of programmed cell death, as well as for persistence of *Salmonella* in the *C. elegans* intestine [17]. Recently Bailey et al. showed that the ability of *Salmonella* Typhimurium to adhere and survive within macrophages is dependent on RamA, a member of the AraC/XylS family of transcriptional regulators. It is also required for colonization in mice and the *C. elegans* intestine [20]. In this study, we constructed a deletion mutant of the multidrug resistance region in DT104, named SNS12, and then assessed the direct involvement of the multidrug resistance (MDR) region of *Salmonella* Typhimurium DT104 in pathogenicity using the *C. elegans* model.

## Materials and Methods

### *C. elegans* and bacterial strains used

*C. elegans* strains N2 (wild type Bristol isolate), SS104 *glp-4* (*bn2*), and *ced-1*(*e1735* and *e1754*) mutant strains were acquired from the *Caenorhabditis* Genetics Center (CGC). All of the strains, except SS104, were maintained at 22°C. SS104 was maintained at 16°C, which is the permissive temperature for this temperature sensitive sterile mutant of *C. elegans*. Strains were cultured in *C. elegans* habitation media (CeHM) in tissue culture flasks on a platform shaker [21]. Nematodes were bleached (0.5 M NaOH, 1% Hypochlorite) to collect eggs, which were incubated in M9 media for 24 hours to bring them to synchronized L1 stage and then transferred to CeHM. To produce synchronized L4 stage worms, L1 worms were grown for an additional 72 hours in CeHM. *Salmonella* Typhimurium DT104 (wild type) and its isogenic ΔMDR mutant, SNS12, (DT104 ∆MDR, this study) and *E. coli* OP 50, a bacterial food strain for *C. elegans*, were used in this study.

### Construction of MDR deletion mutant in DT104

To construct the MDR deletion mutant of DT104, the region, 24,576 to 44,114 bp (GenBank Accession AF261825.2), of SGI1, which contains the 13 kb MDR region, was deleted using two different cloning vectors, pUC19 and pTOPO. The upstream, or left, fragment (24,576 to 26,435 bp) was cloned into pTOPO using primers P1 and P2 (Table 1), containing restriction sites BamHI and PstI (Figure S1). The downstream, or right, fragment (41,379 to 44,114 bp) was cloned into pUC19, using primers P3 and P4 (Table 1), containing restriction sites PstI and HindIII (Figure S1). The upstream fragment was then digested out of the pTOPO vector and ligated into pUC19, which already contained the right fragment. A gene conveying kanamycin resistance, Kan^R^, flanked on both sides by PstI, was digested from plasmid pUC4K (GE Healthcare Life Sciences), and inserted into the PstI site of pUC19 containing the ligated left and right fragments (Figure S1). The whole region, containing the left fragment + Kan^R^ + right fragment, was digested out of pUC19 using BamHI and HindIII enzymes (Figure S1) and cloned into a suicidal vector pMAK705 [22], containing chloramphenicol resistance (Figure S1). The plasmid pMAK705 has a temperature sensitive replicon (pSC101 origin) that is active at 28°C and inactive at 42°C to 44°C. The resulting plasmid was electroporated into the target *Salmonella* Typhimurium DT104 strain and was purified by serial subculture. Through several screening rounds using proper antibiotics and controlled temperature, we isolated the target recombinant and confirmed the exchange of alleles by PCR and measured the Minimum Inhibitory Concentrations (MIC) of different antibiotics (Table 2).

**Table 1:** Primers used in this study.

**Primers**	**Sequence**	**Source**
Primer1	GCGGATCCGCGAACTCTCTATCTCCTCT	This study
Primer2	AAAACTGCAGGACCCGAACTTGATAACTGC	This study
Primer3	AAAACTGCAGCAACCACCATTTCGCAGCAG	This study
Primer4	CCCAAGCTTGTCAAAGCGGTAGCGGAAAC	This study
abf-2(F)	CCATCGTGGCTGCCGACATCGACTTT	[13]
abf-2(R)	GAGCACCAAGTGGAATATCTCCTCCTC	[13]
abu-11(F)	CCAATCGCCCAGTGCCAATCATC	[24]
abu-11(R)	CTGAACATTGGTGTCTCTGTATGG	[24]
pan actin (F)	TCGGTATGGGACAGAAGGAC	[24]
pan actin (R)	CATCCCAGTTGGTGACGATA	[24]
abu-1(F)	CTACTTGCCGAAGCAACAAC	[24]
abu-1(R)	TGGACTGGAGCAGTGTTCTG	[24]
pqn-54b(F)	TCAACCACAACAAACCCAGA	[24]
pqn-54b(R)	GCTTGAGCCTCTTGGATCT	[24]

**Table 2:** Minimum Inhibitory Concentrations (MIC) of the several antibiotics against *Salmonella enterica* serotype Typhimurium phagetype DT104 and its isogenic ΔMDR mutant, SNS12.

**Antibiotic**	**DT104**	**SNS12**
AmiKacin (AMI)	2	2
Ampicillin	>32	2
Amoxicillin /Clavulanic acid (AUG)	16	<=1
Ceftriaxone (AXO)	<=0.25	<=0.25
Cephalothin (CEP)	4	4
Chloramphenicol (CHL)	>32	4
Ciprofloxacin (CIP)	<=0.015	<=0.015
Timethoprim/Sulfamethoxazole (COT)	0.25	<=0.12
Cefoxitin (FOX)	4	2
Gentamycin (GEN)	1	1
Kanamycin (KAN)	<=8	>64
Nalidixic acid (NAL)	4	4
Salfamethoxazole (SMX)	>512	32
Streptomycin (STR)	64	<=32
Tetracycline (TET)	>32	8
Ceftiofur(TIO)	0.5	0.5

### *C. elegans* survival analysis

Survival assays using *C. elegans* were performed following standard protocols [16]. Briefly, bacterial lawns for survival assays were prepared by inoculating 50 µl of an overnight bacterial culture onto Nematode Growth Factor medium (NGM), in 6-cm Petri plates. Plates were incubated overnight at room temperature before the addition of worms. Each experimental group contained 60-80 synchronized worms. Experiments were performed at room temperature, 22°C, except those utilizing SS104, which were performed at 25°C. SS104 [*glp-4 (bn2*)], a temperature sensitive sterile mutant strain of *C. elegans* was employed in several survival assays so as to avoid confounding results due to worm propagation, i.e., second generation worms. Worms were scored every 24 h for survival. Animal survival was plotted using Kaplan-Meier survival curves and analyzed using the Gehan-Breslow-Wilcoxon method to compute P values in GraphPad Prism (GraphPad Software, Inc., La Jolla, CA). Survival assays were repeated at least three times (experiments), with each experiment having three replicates. Results from each experiment were analyzed separately. Survival curves resulting in *p* values of < 0.05 were considered significantly different. Primarily, survival assays were also performed using synchronized **L4** stage SS104 worms; however, some experiments were repeated using other larval stages to confirm and support the results from experiments using N2 worms. For all experiments, *E. coli* OP 50, the normal bacterial food strain, was included as a control.

### Intestinal bacterial count

To assess the degree of colonization of worms by DT104, synchronized L1 stage N2 worms were allowed to feed on either wild type *Salmonella* Typhimurium DT104 or isogenic ΔMDR mutant, SNS12, for 24 hours. Following this exposure, the worms were washed twice with sterile M9 buffer, then placed in NGM media supplemented with gentamicin (25µg/ml) for 20 min to kill bacteria externally attached to the worms. After the worms were washed twice with M9 buffer, they were placed onto OP 50-seeded NGM plates, and incubated for 48 hours. Aliquots of ten worms from each treatment group were collected at time 0, 24hr, and 48hr, and placed in a 2 ml screw cap tube containing 500 µl of sterile M9 buffer with 1% Triton X-100. The worms were mechanically disrupted by using a glass bead beater (Mini-Bead Beater, Biospec Products, Bartlesville, OK). The volume was adjusted with M9 medium to 1 ml and serial dilutions were plated on BHI agar media.

### Microscopy

Live nematodes were mounted on an agar pad on a slide and covered with a cover glass. Sodium azide was used to anesthetize the worms. L1 stage worms were exposed to test bacteria on NGM agar plates for 72 hours at 22°C. Intestinal tracts were examined every 24 hours using a Leica TCS SP5 II confocal microscope (Leica Microsystems, Wetzlar, Germany).

### Transcription level of host genes

Synchronized L1 stage N2 worms were transferred to *C. elegans* habitation media (CeHM) and incubated for 24 hours. The worms were washed with M9 buffer and transferred to NGM plates seeded with DT104 or SNS12 and incubated at 22°C for 72 hours. Worms were collected after 1 hr and after every 24 hours of incubation. The worms were washed in M9 buffer and RNA was extracted using TRIzol (Invitrogen/Life Technologies, Grand Island, NY). Residual genomic DNA was removed by DNase treatment (Turbo DNA-free, Ambion, Austin, TX). Three independent RNA isolations were performed for each experimental treatment for quantitative RT-PCR. cDNA was synthesized from 5 µg of total RNA using random hexamers and SuperScript II reverse transcriptase (Invitrogen/ Life Technologies, Grand Island, NY). Quantitative, or real-time, RT-PCR was performed using SYBR Advantage quantitative PCR premix (Clontech Laboratories, Mountain View, CA) and gene-specific oligonucleotide primers on a Light Cycler (BIO RAD, Hercules, CA). Primers for qRT-PCR are listed in Table 1. Relative fold-change (of exposure to either wild-type DT104 or SNS12 over OP 50) for transcripts was calculated using the comparative *C*_*T*_ (2^−ΔΔCT^) method [23]. Cycle thresholds of amplification were determined by Light Cycler software (BIO RAD, Hercules, CA). All samples were run in triplicate and normalized.

## Results

### Multi Drug Resistance (MDR) phenotype is lost in the MDR deletion mutant of DT104

To test the direct involvement of the MDR region in virulence in *Salmonella* Typhimurium DT104, an isogenic mutant was constructed using allelic exchange (Figure S1). To confirm loss of antibiotic resistance in the ΔMDR isogenic mutant strain of DT104, SNS12, the mutant strain and the wild-type parental strain were assayed for resistance to sixteen antibiotics, using the Kirby-Bauer disk diffusion method (Table 2). The results clearly indicate that DT104 is resistant to ampicillin, amoxicillin, chloramphenicol, streptomycin, sulphamethoxazole, and tetracycline, but the isogenic ΔMDR mutant, SNS12, was resistant only to kanamycin, a result of introduction of the kanamycin resistance gene via allelic exchange (Table 2, Figure S1). It should be noted that generation of the ∆MDR mutation in strain SNS12 did not significantly affect growth rate, compared to the wild-type DT104 parental strain (Figure S2).

### MDR region of *Salmonella* Typhimurium DT104 has virulence properties against *C. elegans*

**Figure 1:**
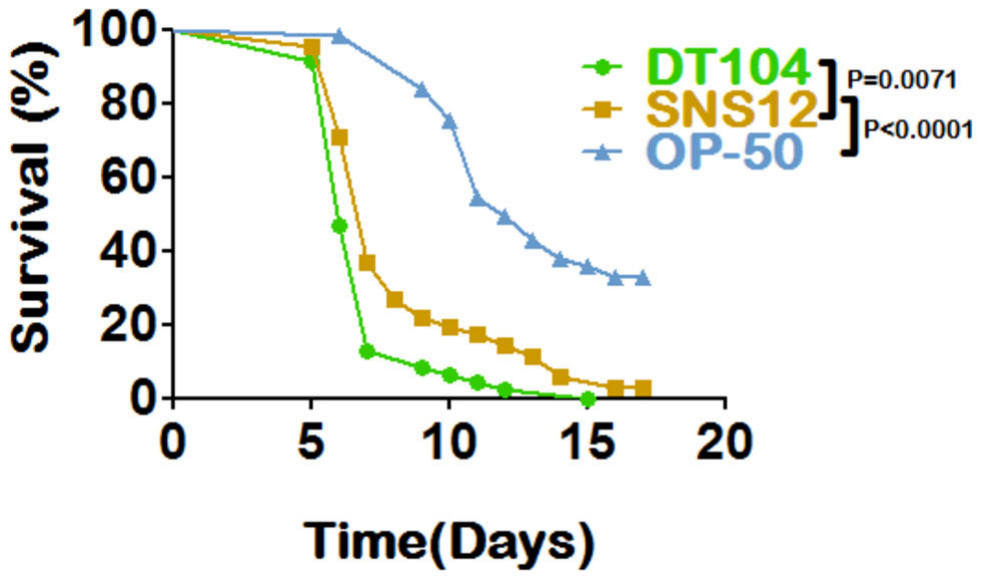
*Salmonella* Typhimurium DT104 kills *C. elegans* significantly faster when MDR genes are present. **L4** stage hermaphrodite **SS104** worms were exposed to wild type *Salmonella* Typhimurium DT104 (--●--),SNS12, a ΔMDR isogenic mutant of DT104 (--■--), and *E. coli* OP 50 (--▲--). P<0.001.

Synchronized L4 stage *C. elegans* worms fed with either wild type DT104 or SNS12 exhibited a significant shortened lifespan (P<0.0001), as compared to worms fed with *E. coli* OP 50 (Figure 1). Further, an attenuated killing response was observed when worms were exposed to SNS12, as compared to worms exposed to wild type DT104 (P=0.0071) (Figure 1), indicating that virulence is enhanced when the MDR region is present. Similar results were observed when other larval stages, such as L1 (Figure S3), or genotypes of *C. elegans* were exposed to DT104 and SNS12 (Table S1). Interestingly, although both larval stages exhibited significantly shortened lifespans when exposed to wild type DT104, L1 stage worms were found to be slightly more sensitive (P<0.0001; Figure S3) than L4 stage worms (Raw data of all independent experiments are shown in Table S1).

### MDR genes induce colonization of *Salmonella* Typhimurium in *C. elegans* intestine

**Figure 2:**
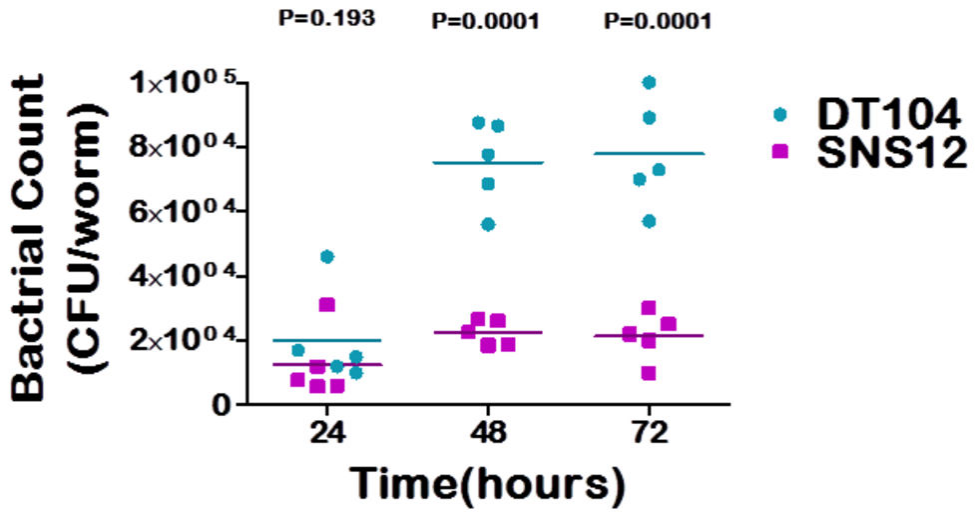
*Salmonella* Typhimurium DT104 colonization in *C. elegans* intestine is enhanced due to MDR genes. **L1** stage **N2** worms were exposed to DT104 (--●--) and SNS12 (--■--), and the extent of colonization was determined every 24 hours. Each data point represents the colony forming unit per worm (CFU worm^-1^) from a pool of 10 infected worms. Horizontal bar indicates the cumulative geometric mean of three independent experiments.

**Figure 3:**
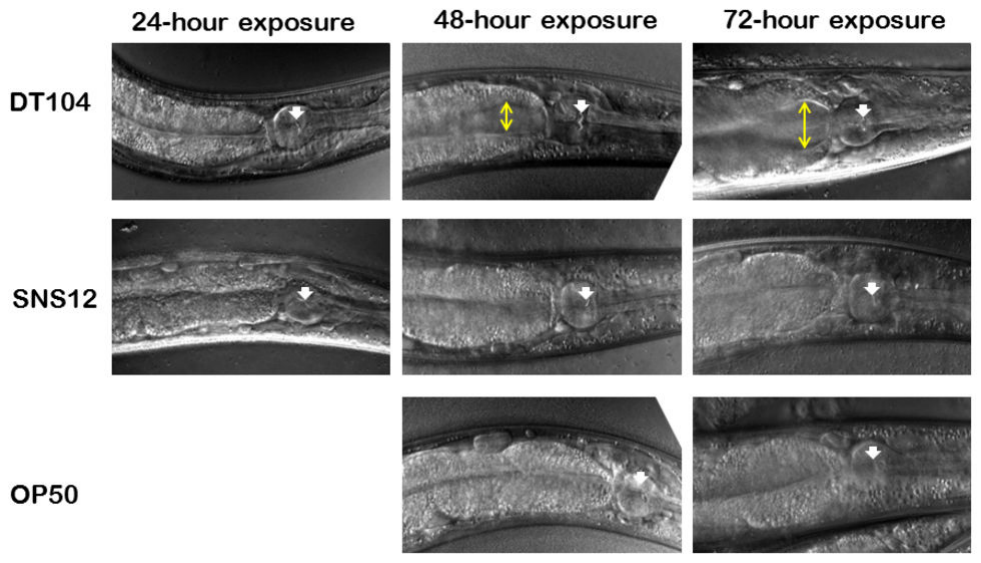
Colonization of the *C. elegans* intestine by *Salmonella* Typhimurium DT104. Confocal microscopy images of representative worms exposed to DT104, SNS12, and *E. coli* OP 50. White arrow shows the grinder of the pharynx, and yellow line shows extent of intestinal distention.

To explore the rate of the intestinal bacterial colonization and proliferation in *C. elegans*, L1 stage wild type N2 worms were exposed to both DT104 and SNS12. After exposure for 24 hours, bacterial colonization of the intestine was on the order of 10^4^ cfu per worm for both bacterial strains (Figure 2). However, as shown in Figure 2, the extent of proliferation of DT104 in the intestine of *C. elegans* is significantly higher (p <0.0001) than that of SNS12 after 48 hours and 72 hours. Nearly identical results were observed when L4 stage SS104 worms were used in the assay (Figure S4). These results indicate that DT104, possessing the MDR locus, exhibits the ability to more efficiently colonize and, more importantly, proliferate in the intestine of *C. elegans*. To confirm the results observed by bacterial plate counts of internalized bacteria, worms were examined using confocal microscopy to determine the extent of intestinal colonization. This is easily achieved for *C. elegans* due to its transparent nature. As compared to SNS12 as well as *E. coli* OP 50, worms exposed to DT104 exhibited significant intestinal distention, indicative of bacterial proliferation (Figure 3).

### *Ced-1* mutants are hyper sensitive to DT104 infection

**Figure 4:**
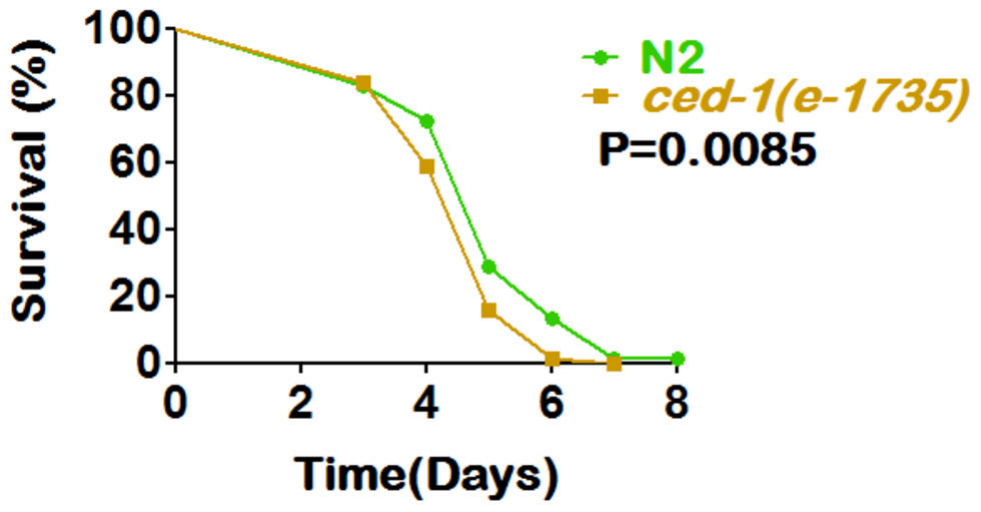
*Ced-1* mutant *C. elegans* worms are more sensitive to killing by DT104. *Ced-1* loss-of-function mutant worms [*ced-1*(e1735)] die significantly faster (P=0.0085) than wild type N2 worms, when exposed to *Salmonella* Typhimurium DT104. **L4** stage wild type **N2** (--●--) and ***ced-1*** (e1735) (--■--) worms were exposed to DT104 and assayed daily for survival.

*C. elegans* has evolved many pathways to recognize microorganisms and to defend itself against infection, and several of these have been identified. For example, loss-of-function mutants of the *ced-1* gene in *C. elegans* are immune compromised and rapidly killed by live bacteria, indicating that it is a component of the innate immune response [24]. Ced-1, homologous to human CD91, a low density lipoprotein receptor, was originally discovered as a component of the programmed cell death pathway, and functions in phagocytic cells to promote cell corpse engulfment [25]. To explore whether the response in *C. elegans* against DT104 harboring the MDR region is *ced-1* dependent, we exposed wild type N2 worms and *ced-1*(*e1735*) mutants to DT104 and and assessed their survival. We found that *ced-1* (*e1735*) mutants die significantly faster (P<0.0085) than wild type worms, when exposed to DT104 (Figure 4) Similar results were observed when an additional *ced-1* mutant *C. elegans* strain, *e1754*, was utilized in the survival assay (Figure S5). These results suggest that the immune response of *C. elegans* to exposure to DT104 is likely mediated through *ced-1*.

### Expression levels of prion-like-(QN-rich)-domain-bearing genes and an antimicrobial peptide (AMP) gene after exposure to *Salmonella* DT104

Ced-1 regulates an alternative Unfolded Protein Response (UPR) pathway, prion-like-(QN-rich)-domain-bearing protein pathway [26], which has a potential role in *C. elegans* innate immunity [24]. Over-expression of prion-like-(QN-rich)-domain-bearing genes is reported to function as a protective response in *C. elegans* against live bacteria, like *Salmonella enterica*-mediated colonization and killing [24]. Additionally, several antimicrobial peptide (AMP) genes, such as *abf-2* and *spp-1*, are expressed in the *C. elegans* pharynx and intestine and have been shown to have antimicrobial activity [13,27–29]. The *abf-2* gene, expressed in the pharynx [28] and homologous to the antibacterial factor ASABF from *Ascaris suum*, has broad activity against a number of gram-positive and gram-negative bacteria, as well as yeast [13,28]. The *spp-1* gene encodes a caenopore, a saponin (B) domain-containing protein, which is a member of the saponin-like protein (SAPLIP) super family. It is expressed in the intestine [30] and exhibits antimicrobial activity against bacterial species such as *Salmonella* Typhimurium and *Pseudomonas aeruginosa* [13,31].

**Figure 5:**
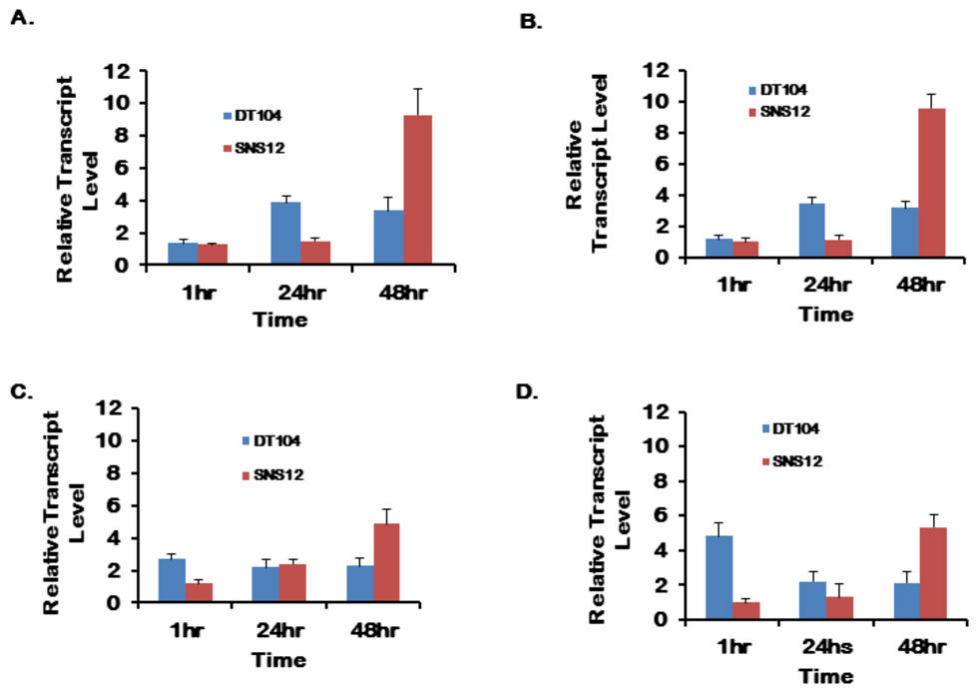
Antimicrobial response to wild type *Salmonella* Typhimurium DT104. Expression of (A) *pqn-54*, (B) *abu-1*, (C) *abf-2* and (D) *abu-11* genes of **N2** worms as determined by qRT-PCR at one hour, 24-hour and 48-hour exposure. Relative expression levels (DT104 or SNS12 over OP 50) are shown as fold changes (mean ± std. err.).

We determined the transcriptional levels of several prion-like-(QN-rich)-domain-bearing genes, namely *pqn-54, abu-1, abu-11*, and antimicrobial peptide (AMP) genes, *abf-2* and *spp-1*, in wild type N2 worms when exposed to DT104. Relative transcription levels of all genes, except *spp-1*, were higher when worms were exposed to either DT104 or SNS12, as compared to *E. coli* OP 50 (Figure 5). We did not find any significant difference in *spp-1* expression levels (data not shown). As for differences between N2 worms exposed to DT104 and SNS12, we observed higher expression in earlier timepoints of exposure, 1 or 24 hours post-exposure, in worms exposed to wild type DT104 (Figure 5). This trend was found to be dependent on intact Ced-1 regulation for gene pqn-54 (Figure S6). Interestingly, this trend is reversed at 48 hours, where expression levels were found to be higher in animals exposed to SNS12 (Figure 5).

## Discussion

Several cohort studies have reported that infection with antibiotic resistant *Salmonella* Typhimurium DT104, harboring the MDR locus, correlated with increased incidence and severity of the disease [7,8]. Inactivation of the MDR region in DT104 has been reported to reduce virulence in chickens [12]. However, results of studies using tissue culture assays and the mouse model of systematic salmonellosis indicate that the enhanced virulence of DT104 is not due to an increase in invasiveness [9–11].

In this study, we found that worms fed DT104 were killed significantly faster than those fed the isogenic ΔMDR mutant, SNS12 (Figure 1), which confirms that antibiotic resistant DT104 is more virulent. Although the difference appears to be only slightly significant, it was highly reproducible, regardless of the genotype and larval stage of worms used (Table S1).

Persistent bacterial infection in the metazoan intestine is an important aspect of pathogenesis, although the underlying molecular mechanism remains to be elucidated. Bacterial growth and proliferation in *C. elegans* intestine specifically has been shown to be an important hallmark of the pathogenic process [16,32]. Colonization of the intestine depends on both the ability of intact bacteria to enter the intestinal lumen [33] and the ability to survive the reaction of the host immune system. When worms are fed with food bacteria, *E. coli* OP 50, they digest almost all and usually no bacteria are found in their intestinal lumen beyond the pharynx. But some pathogenic bacteria, such as *Salmonella* Typhimurium [16], *E. faecalis* [32] and *Vibrio cholerae* (H. Cinar, unpublished data), have been shown to colonize the *C. elegans* intestine. Immune compromised worms are found to be susceptible to bacterial colonization and proliferation, leading to faster killing of worms [34–36]. We found that the rate of bacterial colonization and proliferation is significantly higher (P<0.0001) for wild type DT104 compared to SNS12, the ΔMDR mutant, after 24 to 72 hours (Figures 2 and 3), which indicates that the MDR locus has a significant direct role in *S*. Typhimurium-mediated pathogenesis in *C. elegans*, and that this role is most likely in intestinal proliferation.

Without further genetic investigation, the exact role of the MDR locus in virulence in DT104 isolates can only be speculated. Several possible explanations can be put forward, most of which would involve the presence of a particular protein coding gene within the MDR region. For example, the *floR* gene, which conveys cross-resistance to chloramphenicol and florfenicol encodes a membrane spanning efflux pump protein, which may possess other transport functions [37]. There are also a small number of gene products, namely ORF1, 2, 5, 6, as well as a *groEL* gene fragment, encoded in the MDR region, which currently have unknown functions. Sequence homology points to the possibility of a role in regulation for ORF1 [38]. It is plausible that this gene may be a component of the regulatory network that controls expression of the cytopathic collagenase, *clg*, which encodes SlyA, and possibly other yet unidentified factors [39,40].

In terms of host response, we found a consistently different trend in the expression level of several genes that play a role in innate immune response when exposed to DT104 and SNS12. The Unfolded Protein Response (UPR) signaling system is a protective mechanism which is induced in response to the overload of unfolded proteins in the endoplasmic reticulum; it reestablishes the normal state of the cell by halting protein translation and increasing levels of molecular chaperones involved in protein folding. A non-canonical, UPR signaling, prion-like-(QN-rich)-domain-bearing gene (PQN) pathway, has been found to be responsible for innate immunity in *C. elegans* and regulated in a *ced-1*-dependent manner [24]. Further, the genes, *pqn/abu*, are reported to be over-expressed upon exposure to *S. enterica*, and required for the protection of *C. elegans* against *S. enterica*-mediated killing [24]. In this study, using the *C. elegans* lethality assay, we have shown that *ced-1* mutants were more susceptible to DT104 infection, as compared to wild-type worms, suggesting a role for *ced-1* against MDR-mediated virulence (Figure 4 and Figure S5). Further, we found that the relative expression of *abu-1, abu-11* and *pqn-54* genes are significantly higher at the transcriptional level upon exposure to wild type *S*. Typhimurium DT104 after one hour (*abu-11*) or 24 hours (*pqn-54* and *abu-1*). Conversely, worms exposed to SNS12 showed higher expression of all three genes after 48 hours (Figure 5). The delayed expressions of these genes in worms, after exposure to mutant strain SNS12, suggest that MDR genes in *Salmonella* have significant impact in early immune response. An alternative explanation is the expression, and possibly secretion, of an unidentified factor by antibiotic resistant DT104, which may have an overall affect of repression of these host genes in *C. elegans*. Altogether, our results suggest that the *ced-1* gene and the PQN unfolded protein response pathway are involved in the immune response against exposure to wild type *Salmonella* Typhimurium DT104 in *C. elegans*, specifically during early stages of infection.

Host antimicrobial peptides (AMPs) have an important role in combating pathogenesis. Induction of several antimicrobial peptides is reported in intestinal epithelial-Paneth cells [41–43] and in the intestinal mucosa [44], following bacterial infections, including *Salmonella* Typhimurium. Microorganisms need to overcome AMP-mediated defense to establish persistent infection. Bacterial colonization in the intestine directly correlates with the expression of *C. elegans* antimicrobial peptides, such as, *abf-2* and *spp-1* [13]. We found *abf-2* to be highly expressed in N2 worms against both wild type DT104 and ΔMDR mutant SNS12 exposure, though higher transcript level was observed earlier in DT104 (one hour), compared to SNS12 (48 hours) (Figure 5c). The data suggests that the AMP gene, *abf-2*, but not *spp-1*, is expressed at higher levels by *C. elegans* when exposed to DT104 harboring the multidrug resistance (MDR) region.

Overall, our findings indicate that the multiple drug resistant (MDR) genes of *Salmonella enterica* Typhimurium DT104 directly contribute to the virulence of this organism in *C. elegans*. Specifically, antibiotic resistant DT104, harboring the MDR locus, demonstrated enhanced killing of *C. elegans*, as well as, a much higher level of bacterial colonization and proliferation in the intestinal lumen of worms. We also found that the immune response against this genotype of *Salmonella* acts through the *ced-1* pathway in *C. elegans*. Further, we observed that multiple prion-like-(QN-rich)-domain-bearing and antimicrobial peptide (AMP) genes in *C. elegans* show an immediate or early higher transcriptional response to wildtype DT104, as compared to its ΔMDR isogenic mutant, which further supports the role of the MDR region in virulence in DT104.

## Supporting Information

Figure S1 **Schematic representation of the construction of MDR deletion mutant in DT104 genetic background.**
(TIF)

Figure S2 **Growth curve of DT104 and SNS12 in LB media for 24 hours.**
(TIF)

Figure S3 ***Salmonella* Typhimurium DT104 kills *C. elegans* significantly faster when MDR genes are present.** **L1** stage hermaphrodite **SS104** were exposed to wild type *Salmonella* Typhimurium DT104 (--●--) and SNS12, a ΔMDR isogenic mutant of DT104 (--■--). P<0.0001.
(TIF)

Figure S4 ***Salmonella* Typhimurium DT104 colonization in *C. elegans* intestine is enhanced due to MDR genes.** **L4** stage SS104 worms were exposed to DT104 (--●--) and SNS12 (--■--) and the extent of colonization was determined every 24 hours. Each data point represents the colony forming unit per worm (CFU worm^-1^) from a pool of 10 infected worms. Horizontal bar indicates the cumulative geometric mean of three independent experiments.
(TIF)

Figure S5 ***Ced-1* mutant *C. elegans* worms are more sensitive to killing by DT104.** *Ced-1* loss-of-function mutant worms [*ced-1*(***e1754***)] die significantly faster (P=0.0001) than wild type worms (N2), when exposed to *Salmonella* Typhimurium DT104. L4 stage wild type N2 (--●--) and *ced-1* (***e1754***) (--■--) worms were exposed to DT104 and assayed daily for survival.
(TIF)

Figure S6 **Expression of gene *pqn54* in *C. elegans* upon exposure to *Salmonella* Typhimurium DT104.** Quantitative Real time PCR of **L1** stage **N2** and ***ced-1*** (*e1735*) worms exposed to DT104, SNS12, and OP 50 for 24 hours.
(TIF)

Table S1 **Data and statistical results of replicate *C. elegans* survival assays when exposed to DT104 and SNS12.**
(DOCX)
