# Correction: Identification of Virulence Properties in *Salmonella* Typhimurium DT104 Using *Caenorhabditis elegans*

**DOI:** 10.1371/annotation/10611692-4e13-4c9f-8571-1a69d46628bd

**Published:** 2013-11-07

**Authors:** Surasri N. Sahu, Yuda Anriany, Christopher J. Grim, Sungji Kim, Zenas Chang, Sam W. Joseph, Hediye N. Cinar

The affiliation of the sixth author is incorrect. Sam W. Joseph is not affiliated with institution number 4, but rather with institution number 5, Department of Cell Biology and Molecular Genetics, University of Maryland, College Park, Maryland, United States of America. 

