# Supplementary figures and images for: Identification of Virulence Properties in Salmonella Typhimurium DT104 Using Caenorhabditis elegans
Source: PLoS One. 2013 Oct 4;8(10):e76673. doi: 10.1371/journal.pone.0076673 (PMC3790755; doi:10.1371/journal.pone.0076673)

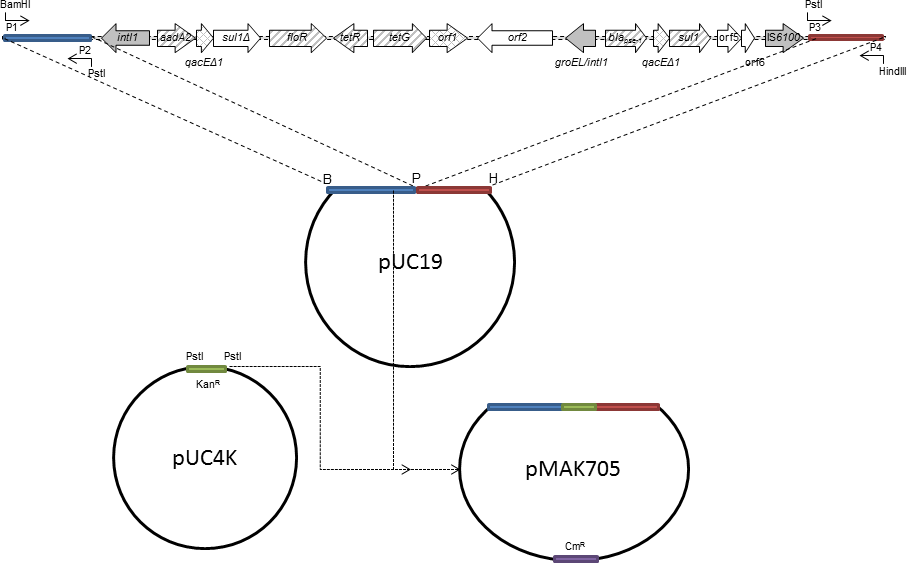

Supplement: Figure S1 — Schematic representation of the construction of MDR deletion mutant in DT104 genetic background. (TIF) [file pone.0076673.s001.tif]

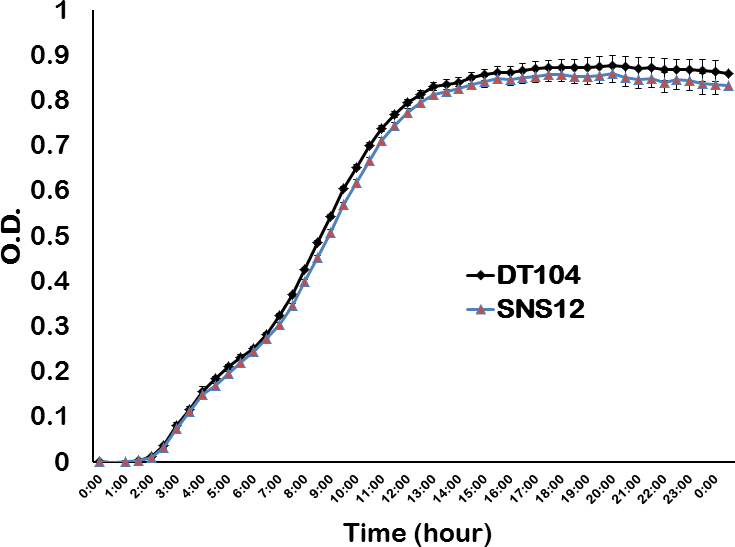

Supplement: Figure S2 — Growth curve of DT104 and SNS12 in LB media for 24 hours. (TIF) [file pone.0076673.s002.tif]

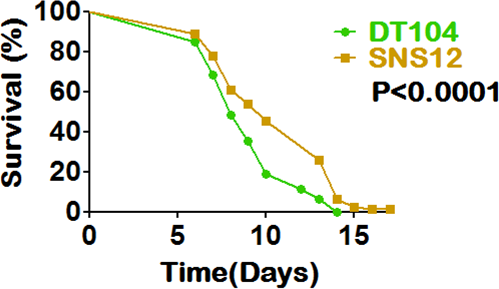

Supplement: Figure S3 — Salmonella Typhimurium DT104 kills C. elegans significantly faster when MDR genes are present. L1 stage hermaphrodite SS104 were exposed to wild type Salmonella Typhimurium DT104 (--●--) and SNS12, a ΔMDR isogenic mutant of DT104 (--■--). P<0.0001. (TIF) [file pone.0076673.s003.tif]

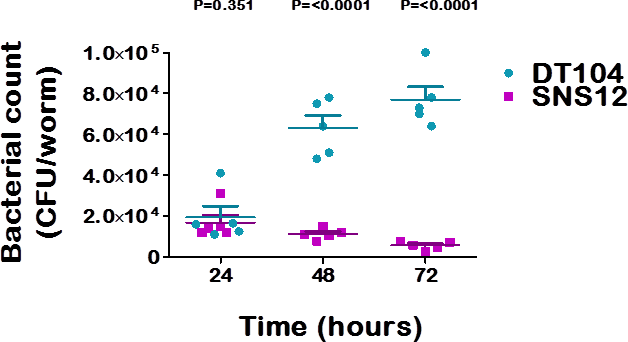

Supplement: Figure S4 — Salmonella Typhimurium DT104 colonization in C. elegans intestine is enhanced due to MDR genes. L4 stage SS104 worms were exposed to DT104 (--●--) and SNS12 (--■--) and the extent of colonization was determined every 24 hours. Each data point represents the colony forming unit per worm (CFU worm-1) from a pool of 10 infected worms. Horizontal bar indicates the cumulative geometric mean of three independent experiments. (TIF) [file pone.0076673.s004.tif]

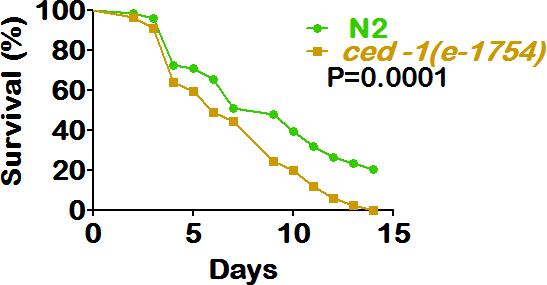

Supplement: Figure S5 — Ced-1 mutant C. elegans worms are more sensitive to killing by DT104. Ced-1 loss-of-function mutant worms [ced-1(e1754)] die significantly faster (P=0.0001) than wild type worms (N2), when exposed to Salmonella Typhimurium DT104. L4 stage wild type N2 (--●--) and ced-1 (e1754) (--■--) worms were exposed to DT104 and assayed daily for survival. (TIF) [file pone.0076673.s005.tif]

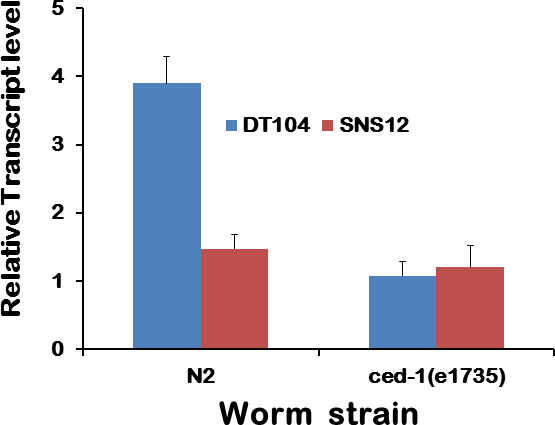

Supplement: Figure S6 — Expression of gene pqn54 in C. elegans upon exposure to Salmonella Typhimurium DT104. Quantitative Real time PCR of L1 stage N2 and ced-1 (e1735) worms exposed to DT104, SNS12, and OP 50 for 24 hours. (TIF) [file pone.0076673.s006.tif]
