# Supplementary material for: Identification of Virulence Properties in Salmonella Typhimurium DT104 Using Caenorhabditis elegans
Source: PLoS One. 2013 Oct 4;8(10):e76673. doi: 10.1371/journal.pone.0076673 (PMC3790755; doi:10.1371/journal.pone.0076673)
Supplement: Table S1 — Data and statistical results of replicate C. elegans survival assays when exposed to DT104 and SNS12. (DOCX) [file pone.0076673.s007.docx]

**Supplementary Table 1.**

Table S1: Data and statistical results of replicate *C*. *elegans* survival assays when exposed to DT104 and SNS12.

| Genotype of worms | Stage of worms | Median survival(days) | | p-value | Number of worms | |
| --- | --- | --- | --- | --- | --- | --- |
|  |  | DT104 | SNS12 |  | DT104 | SNS12 |
| N2 | L4 | 7 | 11 | p=0.0068 | 76 | 89 |
| N2 | L4 | 7 | 9 | p=0.011 | 62 | 59 |
| SS104 | L4 | 6 | 7 | P=0.0071 | 47 | 41 |
| SS104 | L4 | 6 | 7 | P=0.0445 | 54 | 53 |
| SS104 | L1 | 8 | 10 | P=0.0022 | 72 | 78 |
| SS104 | L1 | 8 | 9 | P=0.0098 | 71 | 194 |
